# Unto the third generation: evidence for strong familial aggregation of physicians, psychologists, and psychotherapists among first-year medical and psychology students in a nationwide Austrian cohort census

**DOI:** 10.1186/s12909-017-0921-4

**Published:** 2017-05-03

**Authors:** Ulrich S. Tran, Nina Berger, Martin E. Arendasy, Tobias Greitemeyer, Monika Himmelbauer, Florian Hutzler, Hans-Georg Kraft, Karl Oettl, Ilona Papousek, Oliver Vitouch, Martin Voracek

**Affiliations:** 1 Department of Basic Psychological Research and Research Methods, School of Psychology, University of Vienna, Vienna, Austria; 2 Department of Psychology, University of Graz, Graz, Austria; 3 Department of Psychology, University of Innsbruck, Innsbruck, Austria; 4 Department of Medical Education, Medical University of Vienna, Vienna, Austria; 5 Centre for Cognitive Neuroscience, University of Salzburg, Salzburg, Austria; 6 Division of Cell Genetics, Department of Medical Genetics, Molecular and Clinical Pharmacology, Medical University of Innsbruck, Innsbruck, Austria; 7 Institute of Physiological Chemistry, Medical University of Graz, Graz, Austria; 8 Department of Psychology, University of Klagenfurt, Klagenfurt, Austria

**Keywords:** Familial aggregation, Medical profession, Psychology, Psychotherapy, Undergraduates, Nationwide survey, Gender equity, Austria

## Abstract

**Background:**

Medical students present higher numbers of physician relatives than expectable from the total population prevalence of physicians. Evidence for such a familial aggregation effect of physicians has emerged in investigations from the Anglo-American, Scandinavian, and German-speaking areas. In particular, past data from Austria suggest a familial aggregation of the medical, as well as of the psychological and psychotherapeutic, professions among medical and psychology undergraduates alike. Here, we extend prior related studies by examining (1) the extent to which familial aggregation effects apply to the whole nation-wide student census of all relevant (eight) public universities in Austria; (2) whether effects are comparable for medical and psychology students; (3) and whether these effects generalize to relatives of three interrelated health professions (medicine, psychology, and psychotherapy).

**Methods:**

We investigated the familial aggregation of physicians, psychologists, and psychotherapists, based on an entire cohort census of first-year medical and psychology students (*n* = 881 and 920) in Austria with generalized linear mixed models.

**Results:**

For both disciplines, we found strong familial aggregation of physicians, psychologists, and psychotherapists. As compared with previous results, directionally opposite time trends within disciplines emerged: familial aggregation of physicians among medical students has decreased, whilst familial aggregation of psychologists among psychology students has increased. Further, there were sex-of-relative effects (i.e., more male than female physician relatives), but no substantial sex-of-student effects (i.e., male and female students overall reported similar numbers of relatives for all three professions of interest). In addition, there were age-benefit effects, i.e., students with a relative in the medical or the psychotherapeutic profession were younger than students without, thus suggesting earlier career decisions.

**Conclusions:**

The familial aggregation of physicians, psychologists, and psychotherapists is high among medical and psychology undergraduates in Austria. Discussed are implications of these findings (e.g., gender equity, feminization of the medical field, ideas for curricular implementation and student counselling), study limitations, and avenues for future research.

**Electronic supplementary material:**

The online version of this article (doi:10.1186/s12909-017-0921-4) contains supplementary material, which is available to authorized users.

## Background

The medical profession runs in families, as has been shown for English-speaking countries (USA [1–3]; UK [4–7]; New Zealand [8]), Scandinavia (Norway [9]), and German-speaking countries (Germany [10]; Austria [11]). Medical students report far higher proportions of relatives in the medical profession than can be expected from the overall prevalence of physicians in the total population (for a review of the literature on this topic and empirical findings therefrom, see [11]). Proportions range from 13 to 25% for any medically qualified parent [4–10], and from 30% [8] to 46% [11] for any medically qualified relative. In comparison (extrapolated from the latest WHO figures; see [12]), only 0.3% of the total US and UK populations are physicians, and numbers are similar for other industrialized Western nations. Proportions of medical relatives are higher for male than female students [5, 9, 11], higher for first-degree than for more distant relatives [6, 10, 11], higher for male than for female relatives [6, 8, 11], and, on the whole, such effects may have further increased in recent years [4, 5].

The familial aggregation of physicians and apparent sex-of-student and sex-of-relative effects are interesting for a number of reasons. First, it is noteworthy that such an extent of familiality of the profession has not been found for other academic fields [13]. Second, having a medical family background appears to be beneficial for medical students regarding admission rates [7], study drop out [2, 4, 5], and study progress [2, 11] (but see [14]). Thus, familial aggregation appears to boost overall academic success in medicine. Third, the medical profession has high social prestige and status in the general population [15]. At the same time medicine still is perceived as a male profession, both historically and with regards to current obstacles, such as family demands that delay or prevent women’s careers, and thus gender equity, in the medical field (e.g., [16–19]). Women are also underrepresented among the most prestigious medical specialties, such as surgery [19]. However, a feminization of medicine has been noted in recent years [20], and differences in specialty preferences appear to level off, at least in some European countries (see [21] for data from Sweden). International studies on the familial aggregation of physicians among medical students may therefore not only shed light on the gender gap in medicine, but may also serve to highlight and comprehend temporal changes and national differences in the phenomenon under scrutiny.

Extending previous work, Voracek et al. [11] presented evidence that the familial aggregation of physicians is not limited to medical students, but is also present, albeit to a lesser extent, among psychology students. Also, psychology students display a familial aggregation of psychologists and psychotherapists. Psychology, especially its clinical and health-oriented subdisciplines, and psychotherapy are professions that have close relations and overlap areas to some medical specialties, like psychiatry, neurology, pediatrics or geriatrics.

Drawing on prior related findings, as reviewed above, the current study presents data on the familial aggregation of physicians, and of psychologists and psychotherapists, among first-year students of medicine and psychology from all eight Austrian public universities, by nationwide census data of the respective student cohorts of the academic year 2011/12. In particular, we were interested in examining the extent to which previously reported familial aggregation effects (1) would apply to the whole nation-wide student census of a German-speaking Central European country; (2) whether such familial aggregation effects are comparable for medical and psychology students; (3) and whether these effects generalize to relatives of three interrelated health professions (medicine, psychology, and psychotherapy). Having a professionally qualified relative in one’s own academic field of study conceivably has ramifications on study choice and study success, and may also help in explaining the gender gap in medicine (see above). Answering these interrelated research questions may therefore increase insight into these important issues. Moreover, this may also entail implications for curricular implementation, by identifying specific needs of students without a professional family background. Previous research mostly did not investigate nationwide student cohorts (for exceptions, see [4, 5, 9]), let alone census data. Extant data from Austria [11] were limited to the capital city’s public universities (University of Vienna and Medical University of Vienna). The current data thus allowed for the first time a detailed and representative look into the patterns of these familial associations, encompassing first- to third-degree relatives, based on a nationwide census of one cohort of medical and psychology first-year students in Austria. Among other research hypotheses addressed, the present study also serves as a suitable follow-up to earlier data from Austria [11], in testing whether previously reported associations still hold after 12 years.

We expected to replicate the strong familial aggregation of medical relatives among both medical and psychology students, and the familial aggregation of psychologists and psychotherapists specifically among psychology students. Further, we expected sex-of-student and sex-of-relative effects, as evidenced in previous studies. Also, lateral family effects (i.e., patrilateral/father-side vs. matrilateral/mother-side effects) were investigated for the first time. In the absence of previous data, we had no directional expectations with regards to these additional effects. Lastly, drawing on a sample of senior medical students, Voracek et al. [11] reported that men with a medical family background were younger, which age difference may reflect quicker study progress. Alternatively, these students could also have made the decision to study medicine already at an earlier age. We therefore examined whether a medical family background was associated with the age of first-year students, and also tested for similar family background effects for the psychological and psychotherapeutic professions. A significant proportion of students in Austria are foreign citizens (according to national authorities, around 23% of all university students in the winter term 2011/12, of which subgroup 38% are Germans; see [22]). Therefore, we also explored, and controlled for, effects of non-Austrian nationality on overall rates of familial aggregation and on students’ age.

## Methods

### Participants and procedure

This study used a representative sampling frame. The sample was the entire (nationwide) cohort of medical and psychological first-year students at all (eight) relevant Austrian public universities and thus effectively a census of this population. Altogether, data of 881 medical students (46.9% women; ages ranging from 18 to 40 years, *M* = 20.65, *SD* = 2.31) and 920 psychology students (73.4% women; ages ranging from 18 to 65 years, *M* = 22.13, *SD* = 4.94) were used in this study. Medical students were from all three Austrian public medical universities: Vienna (74.0%), Innsbruck (13.4%), and Graz (12.6%). Psychology students were from all five applicable Austrian public universities (i.e., psychology study locations): Vienna (54.1%), Graz (16.0%), Innsbruck (10.5%), Klagenfurt (10.0%), and Salzburg (9.3%). Incidentally, to the best of our knowledge, this investigation constitutes a first-time instance of a research collaboration assembling investigators from the entirety of the three medical and the five psychology study locations existing at public universities in Austria on a joint research theme.

Overall, 37.7% of respondents (28.4% in medicine, 46.6% in psychology) had a non-Austrian nationality: within this group, the majority was of German nationality (32.1%), followed by Turkish (2.8%), and Italian (0.7%; the remaining 2.1% were mostly of European descent). Therefore, henceforth we refer to non-Austrian respondents also as “German/other”. The proportions of non-Austrian students differed between universities: for medicine, proportions were lowest in Graz (22.5%) and highest in Innsbruck (39.8%); for psychology, proportions were lowest in Graz (20.4%) and highest in Salzburg (89.5%).

All data were collected in compulsory courses and introductory lectures for second-term students. In Austria, admission to the studies of medicine and psychology is regulated via admission tests since 2005. Applicants passing these tests are admitted to study, but have to pass a number of exams during the first few semesters in an introductory phase, in order to attend the more advanced courses and lectures. In selecting the cohort of students in the second semester, we ensured that early dropouts (i.e., students dropping out already in the first semester of the introductory phase) did not influence results.

Per institution, data collection took place on a single testing day (in summer term 2012) among seminar groups or lecture audiences. Data collection was direct, group-based, without advance notice, and took between 2 to 5 min. Individuals volunteered to participate in this research; no incentives or rewards were provided. Nonparticipation in the study was mainly due to course absenteeism on the testing day. Table 1 provides detailed information on the characteristics of the data collection, demographic information of the participants, and response rates and coverage rates per study site. Overall, groups comprised around 10 (for courses or seminar groups) to 147 students (for lectures). Response rates ranged from 97% to 100%. Accounting for absent students, overall coverage rates were 88.7% for medicine and 77.2% for psychology. From their own accounts, 98.2% of medical students were in their second, 0.7% in their first, and 1.1% in their third to eighth semester; 81.7% of psychology students were in their second, 1.1% in their first, and 17.2% in their third to sixteenth semester (with *Mdn* = 4 semesters for this latter subgroup).

**Table 1:** Characteristics of data collection, participant demographic information, response rate, and coverage rate per study site

Study site	Local study site collaborator	Course type	*n*	% women	Age in years *M* (*SD*)	Response rate (in %)	Coverage rate (in %)
Medicine							
Vienna	Monika Himmelbauer	All 74 parallel groups (maximally 10 students each) of one compulsory introductory seminar	652	44.6	20.7 (2.5)	97.0	88.1
Graz	Karl Oettl	Introductory lecture	111	56.8	20.9 (2.2)	98.0	88.8
Innsbruck	Hans-Georg Kraft	Introductory lecture	118	50.0	20.3 (1.6)	99.0	92.4
Total			881	46.9	20.7 (2.3)	97.4	88.7
Psychology							
Vienna	Martin Voracek, Ulrich S. Tran	All 15 parallel groups (maximally 40 students each) of one compulsory introductory course	498	73.1	21.8 (3.6)	98.0	92.6
Graz	Ilona Papousek	Introductory lecture	147	76.2	21.9 (5.2)	97.0	63.9
Klagenfurt	Oliver Vitouch	Introductory lecture	92	80.4	26.5 (9.7)	99.0	80.0
Salzburg	Florian Hutzler	Introductory lecture	86	76.7	21.0 (2.3)	98.0	43.0
Innsbruck	Tobias Greitemeyer	Introductory lecture	97	60.8	20.3 (1.6)	100.0	89.0
Total			920	73.4	22.1 (4.9)	98.2	77.2

### Instruments

Data were collected with a one-sheet survey form (see Additional file 1), asking respondents about their demographics (sex, age, nationality, and semester) and whether the following relatives either had completed, or currently studied, academic studies of medicine or psychology, or had completed, or currently underwent, psychotherapeutic training: sisters and brothers (including half-siblings), mother, father, grandparents, aunts and uncles, and parents’ siblings-in-law (separately for male and female relatives, and separately for paternal and maternal relatives). The survey form did not allow ticking multiple occurrences of physicians, psychologists or psychotherapists for the same category of relative; instead, such multiple occurrences were only counted once (see Additional file 1). Occurrences of multiple professions for one type of relative (e.g., physician and psychologist ticked off for the relative category of ‘sister’) were assigned to all respective categories.

### Statistical analysis

The percentages of relatives in the professions of medicine, psychology, and psychotherapy are presented overall as well as separately for medical and psychology students and separately for men and women. Following Voracek et al. [11], we further report the relative risk (*RR*; rounded to the nearest integer) for students of medicine and psychology having a relative in one of the three professions of interest. Accordingly, we calculated the ratio of the percentage of medical (or other) relatives in the student samples (as the “exposed group”) to the percentage of physicians (or the other professions) in the total population for Austria (as the “control group”). These latter population figures were ascertained from official Austrian sources [23–28]. In cases where data for 2012 were missing, data for 2011 or 2010 were used instead. Population figures amounted to 0.64% for physicians (including students of medicine), 0.29% for psychologists (including students of psychology), and 0.16% for psychotherapists (including psychotherapists in training). The *RR*s compared the prevalence rates among the student samples (in the numerator) to the corresponding prevalence rates in the total population (in the denominator). As the latter group was not another sample, but rather the total Austrian population and thus disparately larger, calculation and utilization of confidence intervals for the *RR* values was waived.

Generalized linear mixed models were then utilized to analyze the patterns of familial aggregation in SPSS 21. The generalized linear mixed model is an extension of the general linear model (e.g., ANOVA) that allows for the modeling of non-normally distributed dependent variables and of within-subject data designs [29]. In this model class, the dependent variable may follow a wide range of non-normal distributions, for example, the Bernoulli distribution (dichotomous variable), the binomial or Poisson distributions (count variable), or the gamma distribution (positively skewed continuous variable). In addition, generalized linear mixed models are characterized by the use of link functions which link the linear combination of the predictor variables with the mean of the outcome variable. Such link functions are familiar from more widely used models, such as logistic regression, wherein the logit function serves as the link function.

In the present study, relatives’ affiliations to the professions of medicine, psychology or psychotherapy were coded dichotomously (0 = *no*, 1 = *yes*) and therefore the distributions of these variables were modeled with Bernoulli distributions. The logit function was utilized as the link function. This type of analysis may be thought of as a variant (or extension) of the customary multivariate logistic regression model, with additional allowance for within-subject factors. Like multivariate logistic regression analysis, this type of analysis provides effect estimates in the form of odds ratios that are adjusted for all the other factors in the model. We report both odds ratios (with their 95% confidence intervals) and unstandardized regression weights (*b*s; along with their standard errors); exponentiation of these weights yields the odds ratios. To model the within-subject factors, a covariance matrix is required in the generalized linear mixed model. The covariance matrix was estimated with robust methods from the data. To provide robust significance tests, the Satterthwaite approximation to the degrees of freedom was used.

The first model combined medical students and psychology students, first- to third-degree relatives, and relatives from all three professions of interest in a single multivariate analysis. It analyzed effects of nationality of the respondent (German/other vs. Austrian), field of study (psychology vs. medicine), respondent sex (men vs. women), degree of relatedness to relative (third vs. second vs. first), and profession of relative (psychotherapy vs. psychology vs. medicine) on familial aggregation. Degree of relatedness and profession of relative were modeled as within-subject factors, and nationality, field of study, and respondent sex as between-subject factors. First-degree relatives comprised parents and siblings, second-degree relatives comprised grandparents and aunts and uncles, and third-degree relatives comprised the cousins of the respondents and parents’ siblings-in-law. For the sake of increasing the stability of parameter estimation and model parsimony, only the (contentwise relevant) interaction terms of (respondent sex) × (degree of relatedness), (respondent sex) × (profession of relative), and (field of study) × (profession of relative) were included in the model. From all possible interactions, only these were of substantive interest, as they included factors that have proved important in prior related research. As there was no reason to assume nationality to be an effect moderator, only the main effect of nationality was investigated and controlled for in this analysis.

Subsequent generalized linear mixed models analyzed effects of nationality, respondent sex, sex of relative (men vs. women), generation (elder vs. younger [among first-degree relatives: parents vs. siblings; among second-degree relatives: grandparents vs. aunts/uncles; among third-degree relatives: parents’ siblings-in-law vs. respondents’ cousins]), and laterality (paternal vs. maternal; among second-degree and third-degree relatives only) on having a relative specifically in the medical profession among medical students (separately for first-degree, second-degree, and third-degree relatives). For comparison, similar analyses (reported in Additional file 2) were also performed among psychology students to inquire whether patterns of medical family background were specific for medical students or generalized to psychology students as well. Further models analyzed the effects of the above-mentioned factors on having a relative in the psychological or psychotherapeutic professions among psychology students and, for comparison, among medical students as well (reported in Additional file 2). In these analyses, only patterns of first-degree relatives were investigated, because the sparseness of cell numbers otherwise would have made analyses unstable and unfeasible.

In all of the above subsequent analyses, sex of relative, generation, and laterality were modeled as within-subject factors, and nationality and respondent sex as between-subject factors. Because of data sparseness, and because only two-way interactions were of substantive interest (see above), no three-way interactions were tested in the models for second-degree and third-degree relatives. Data sparseness further prohibited investigating the three-way interaction in the analysis of first-degree relatives in the psychological profession, and the three-way interaction, and interactions of (respondent sex) × (generation) and (generation) × (sex of relative) in the analysis of first-degree relatives in the psychotherapeutic profession among medical students. As we did not suspect nationality to be an effect moderator, only the main effect of nationality was investigated and controlled for in these analyses.

Effects of a medical familial background on respondent age were investigated with generalized linear models separately for medical and psychology students, testing effects of background (first-degree relative), while controlling for respondent sex and nationality in a full factorial model, including also the number of semesters (i.e., academic age) as a covariate. In these analyses, gamma distributions were used to model respondent age. The gamma distribution is a continuous probability distribution of which the exponential and the chi-squared distribution are special cases. Its use allowed to adequately model the positive skew apparent in the distribution of respondent age in the data (medical students: skewness = 2.62; psychology students: skewness = 4.22). Effects of a familial background in psychology and psychotherapy on respondent age were analyzed in a similar way. In contrast to the above analyses, this type of analysis did not yield odds ratios for effect estimates, as the dependent variable was continuous, rather than dichotomous. Just like in ordinary ANOVA, mean differences (and their standard errors) are reported.

### Open science practices

In the spirit of Open Science practices and reproducible research [30, 31], we report how we determined our sample size, all data exclusions (if any), all manipulations, and all measures in the study [32]. Specifically, as our study was a complete survey (namely, a nationwide census of a well-defined cohort), the sample size available for analysis can be considered as simply obtained (or arrived at) rather than having been determined. The dataset supporting the conclusions of this article was available for peer review and is available from the corresponding author on reasonable request. We have opted for not making the dataset publicly available, because it originates from a nationwide cohort census conducted in a comparatively small country, with a high response rate, known time of data collection, and two stratification variables (city/university and discipline). The underlying population is finite and well-delineated, thus implying that individual respondents potentially might be identifiable. We did not exclude any of the received data. Owing to the study design, there were no experimental (or other) manipulations, and the study did not comprise further variables beyond those contained in the one-page survey form (made accessible in Additional file 1). One investigator (author N.B.) travelled to the eight study sites located in five Austrian cities (Graz, Innsbruck, Klagenfurt, Salzburg, and Vienna) and, in coordination with the respective local study site collaborators (see author affiliations and the corresponding information in Table 1), secured the data on-site. We did not preregister a study protocol for this investigation, but have made accessible the original (German-language) survey sheet (Additional file 1). Thus formally this study earns one of the three Open Practices Badges (Open Materials Badge, but not the Preregistration and Open Data Badges; see [33, 34]).

## Results

### Familial aggregation of professions: overall pattern of associations and risk ratios

**Fig. 1:**
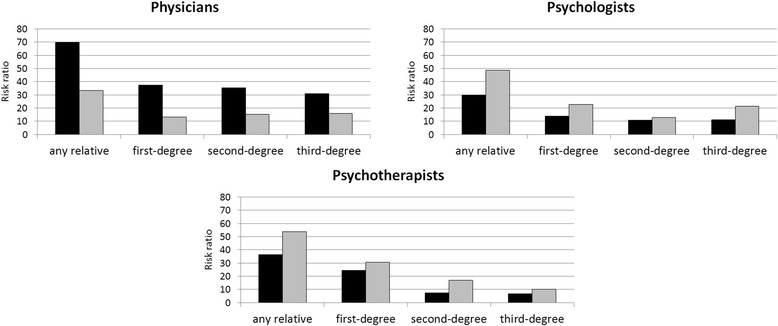
Risk ratios for familial aggregration. Risk ratios for the familial aggregation of physicians, psychologists, and psychotherapists among medical students (*black*) and psychology students (*gray*), comparing the prevalence rates of these three professions among the relatives of medical and psychology students to the prevalence of the same professions in the total Austrian population

Descriptive statistics of the familial aggregation of relatives in the medical, psychological, and psychotherapeutic professions are presented in Table 2 (medical students) and Table 3 (psychology students). Risk ratios of the familial aggregation of physicians, psychologists, and psychotherapists were sizeable for both groups. For a better understanding and to facilitate comparisons, *RR*s are additionally visualized in Fig. 1. The familial aggregation of physicians was more pronounced among medical students (any relative: *RR* = 44.6%/0.64% = 70; first-, second-, and third-degree relatives: *RR* = 38, 35, and 31) than among psychology students (any relative: *RR* = 21.3%/0.64% = 33; first-, second-, and third-degree relatives: *RR* = 13, 15, and 16), whereas familial aggregation of psychologists and psychotherapists was more pronounced among psychology students (psychologists: any relative: *RR* = 14.1%/0.29% = 49; first-, second-, and third-degree relatives: *RR* = 23, 13, and 21; psychotherapists: any relative: *RR* = 8.6%/0.16% = 54; first-, second-, and third-degree relatives: *RR* = 31, 17, and 10) than among medical students (psychologists: any relative: *RR* = 8.7%/0.29% = 30; first-, second-, and third-degree relatives: *RR* = 18, 11, and 11; psychotherapists: any relative: *RR* = 5.8%/0.16% = 36; first-, second-, and third-degree relatives: *RR* = 24, 8, and 7).

**Table 2:** Familial aggregation of physicians, psychologists, and psychotherapists among medical students

Type of relationship to respondent	Total (*N* = 881)			Men (*n* = 468)			Women (*n* = 413)		
	Med	Psy	Pt	Med	Psy	Pt	Med	Psy	Pt
First-degree relatives									
Father	19.6	0.5	2.2	20.9	0.9	2.6	18.2	0.0	1.7
Mother	6.4	1.1	1.6	5.6	1.7	1.1	7.3	0.5	2.2
Brother	1.9	0.3	0.1	2.1	0.4	0.2	1.7	0.2	0.0
Sister	3.2	2.5	0.2	3.0	2.6	0.4	3.4	2.4	0.0
Second-degree relatives^a^									
Grandfather	5.7/5.9	0.3/0.3	0.2/0.2	6.6/5.3	0.4/0.6	0.4/0.0	4.6/6.5	0.2/0.0	0.0/0.5
Grandmother	2.4/1.7	0.1/0.1	0.0/0.0	2.4/1.5	0.2/0.2	0.0/0.0	2.4/1.9	0.0/0.0	0.0/0.0
Uncle	5.7/5.3	0.2/0.2	0.0/0.2	5.6/4.7	0.2/0.4	0.0/0.0	5.8/6.1	0.2/0.0	0.0/0.5
Aunt	4.5/3.2	0.9/1.0	0.2/0.3	4.9/3.0	0.4/0.6	0.2/0.2	4.1/3.4	1.5/1.5	0.2/0.5
Third-degree relatives^a^									
Parents’ brother-in-law	3.2/3.3	0.1/0.5	0.1/0.1	4.3/2.8	0.0/0.4	0.0/0.0	1.9/3.9	0.2/0.5	0.2/0.2
Parents’ sister-in-law	2.8/2.2	0.6/0.2	0.3/0.1	2.6/1.7	0.4/0.0	0.2/0.0	3.1/2.7	0.7/0.5	0.5/0.2
Male cousin	4.5/2.4	0.1/0.2	0.0/0.1	3.8/2.1	0.0/0.0	0.0/0.2	5.3/2.7	0.2/0.5	0.0/0.0
Female cousin	4.4/5.6	0.7/0.9	0.3/0.1	3.0/4.9	0.4/0.4	0.2/0.0	6.1/6.3	1.0/1.5	0.5/0.2
Any first-degree relative	24.0	4.0	3.9	24.8	5.1	4.1	23.0	2.7	3.6
Any second-degree relative	22.7	3.1	1.2	22.0	2.8	0.9	23.5	3.4	1.7
Any third-degree relative	19.8	3.2	1.1	17.9	1.7	0.6	21.8	4.8	1.7
Any relative	44.6	8.7	5.8	44.4	8.8	5.3	44.8	8.7	6.3

Numbers are percentages. *Med* physicians and medical undergraduates combined, *Psy* psychologists and psychology undergraduates combined, *Pt* psychotherapists and individuals in psychotherapeutic training combined. ^a^Father’s/mother’s side

**Table 3:** Familial aggregation of physicians, psychologists, and psychotherapists among psychology students

Type of relationship to respondent	Total (*N* = 920)			Men (*n* = 245)			Women (*n* = 675)		
	Med	Psy	Pt	Med	Psy	Pt	Med	Psy	Pt
First-degree relatives									
Father	5.2	1.7	1.3	5.3	1.6	3.3	5.2	1.8	0.6
Mother	2.3	3.3	3.4	1.6	3.3	2.9	2.5	3.3	3.6
Brother	2.2	0.8	0.3	3.3	1.2	0.4	1.8	0.6	0.3
Sister	1.1	1.4	0.4	1.2	2.0	0.4	1.0	1.2	0.4
Second-degree relatives^a^									
Grandfather	1.2/1.2	0.1/0.1	0.2/0.0	1.2/1.2	0.4/0.4	0.0/0.0	1.2/1.2	0.0/0.0	0.3/0.0
Grandmother	0.8/0.8	0.0/0.4	0.2/0.1	0.0/0.8	0.0/0.8	0.4/0.0	1.0/0.7	0.0/0.3	0.1/0.1
Uncle	1.5/3.8	0.7/0.7	0.2/0.8	0.8/3.3	0.8/0.8	0.0/1.2	1.8/4.0	0.6/0.6	0.3/0.6
Aunt	1.2/2.2	1.0/1.1	0.3/1.0	1.2/2.4	0.0/1.6	0.4/1.2	1.2/2.1	1.3/0.9	0.3/0.9
Third-degree relatives^a^									
Parents’ brother-in-law	1.2/2.0	0.3/0.4	0.2/0.0	0.8/1.2	0.0/0.4	0.4/0.0	1.3/2.2	0.4/0.4	0.1/0.0
Parents’ sister-in-law	1.2/1.1	0.3/0.7	0.3/0.1	0.8/1.2	0.0/0.0	0.8/0.0	1.3/1.0	0.4/0.9	0.1/0.1
Male cousin	0.8/2.1	0.2/0.3	0.3/0.1	0.4/1.2	0.0/0.4	0.4/0.0	0.9/2.4	0.3/0.4	0.3/0.1
Female cousin	1.8/2.9	1.6/2.5	0.3/0.3	1.6/2.4	1.6/2.4	0.0/0.8	1.9/3.1	1.6/2.5	0.4/0.1
Any first-degree relative	8.5	6.6	4.9	8.2	8.2	5.3	8.6	6.1	4.7
Any second-degree relative	9.8	3.7	2.7	7.8	4.1	2.9	10.5	3.6	2.7
Any third-degree relative	10.3	6.2	1.6	8.6	4.9	2.0	11.0	6.7	1.5
Any relative	21.3	14.1	8.6	18.8	15.5	9.4	22.2	13.6	8.3

Numbers are percentages. *Med* physicians and medical undergraduates combined, *Psy* psychologists and psychology undergraduates combined, *Pt* psychotherapists and individuals in psychotherapeutic training combined. ^a^Father’s/mother’s side

Table 4 lists the results of the first model that combined medical students and psychology students, first- to third-degree relatives, and relatives from all three professions of interest into a single analysis. Overall, familial aggregation was slightly more pronounced among respondents with other than Austrian nationality and more pronounced among medical than psychology students. Respondents overall were more likely to report relatives from the medical profession than from the psychology and psychotherapy professions. However, psychology students were more likely than medical students to report relatives in the professions of psychology and psychotherapy (field of study × profession-of-relative interaction). Further, whereas percentages of relatives in the professions of interest remained relatively stable across first- to third-degree relatives among women (see Tables 2 and 3), numbers declined among men for third-degree relatives, as compared to first-degree relatives (respondent sex × degree-of-relatedness interaction). For second-degree relatives, a similar decline was observed among men; however, the respective interaction was not nominally significant (*p* = .058).

**Table 4:** Effects of nationality, field of study, respondent sex, profession of relative, and degree of relatedness to relative on the familial aggregation of physicians, psychologists, and psychotherapists among medical and psychology students

Effect	*b* (*SE*)	*OR* [95% confidence interval]
Nationality (German/other vs. Austrian)	0.17 (0.08)*	**1.19 [1.01–1.40]**
Field of study (psychology vs. medicine)	−1.07 (0.10)***	**0.34 [0.28–0.42]**
Respondent sex (men vs. women)	0.12 (0.12)	1.13 [0.89–1.43]
Degree of relatedness (third vs. first)	−0.004 (0.10)	1.00 [0.83–1.20]
(second vs. first)	−0.07 (0.09)	0.94 [0.78–1.12]
Profession of relative (psychology vs. medicine)	−2.14 (0.17)***	**0.12 [0.08–0.16]**
(psychotherapy vs. medicine)	−2.82 (0.21)***	**0.06 [0.04–0.09]**
Respondent sex (men) × degree of relatedness (second)	−0.27 (0.14)	0.76 [0.58–1.01]
Respondent sex (men) × degree of relatedness (third)	−0.52 (0.16)**	**0.60 [0.44–0.81]**
Respondent sex (men) × profession of relative (psychology)	0.09 (0.18)	1.09 [0.77–1.56]
Respondent sex (men) × profession of relative (psychotherapy)	0.09 (0.23)	1.09 [0.70–1.71]
Field of study (psychology) × profession of relative (psychology)	1.51 (0.19)***	**4.53 [3.16–6.51]**
Field of study (psychology) × profession of relative (psychotherapy)	1.46 (0.23)***	**4.32 [2.77–6.73]**

Only non-redundant model parameters are presented here. With regards to investigated effects, only interactions of substantive interest were included in the models (see main text)

Significant effects (*p* < .05) are printed boldface *p <. 05, **p < .01, ***p < .001

### Relatives in the medical profession

Among medical students (Table 5), first-degree relatives in the medical profession were more likely parents than siblings, and, among parents, more likely fathers than mothers (generation × sex-of-relative interaction). The effect of generation was also observable among psychology students (Additional file 2: Table S1), but not the interaction.

**Table 5:** Effects of nationality, respondent sex, sex of relative, generation, and laterality (among second-degree and third-degree relatives) on the familial aggregation of physicians among medical students, separately for first-degree, second-degree and third-degree relatives (includes parents’ siblings-in-law)

Effect	*b* (*SE*)	*OR* [95% confidence interval]
*First-degree relatives*		
Nationality (German/other vs. Austrian)	0.23 (0.17)	1.26 [0.90–1.74]
Respondent sex (men vs. women)	−0.13 (0.38)	0.88 [0.41–1.86]
Generation (parents vs. siblings)	0.80 (0.32)*	**2.23 [1.20–4.15]**
Sex of relative (men vs. women)	−0.71 (0.45)	0.49 [0.20–1.19]
Respondent sex (men) × generation (parents)	−0.16 (0.45)	0.85 [0.35–2.08]
Respondent sex (men) × sex of relative (men)	0.37 (0.58)	1.44 [0.46–4.51]
Generation (parents) × sex of relative (men)	1.75 (0.46)***	**5.77 [2.32–14.32]**
Respondent sex (men) × generation (parents) × sex of relative (men)	0.10 (0.63)	1.10 [0.32-3.80]
*Second-degree relatives*		
Nationality (German/other vs. Austrian)	0.28 (0.16)	1.33 [0.97–1.81]
Respondent sex (men vs. women)	−0.27 (0.28)	0.77 [0.44–1.33]
Laterality (paternal vs. maternal)	0.20 (0.26)	1.22 [0.74–2.02]
Generation (grandparents vs. aunts/uncles)	−0.66 (0.26)*	**0.52 [0.31–0.86]**
Sex of relative (men vs. women)	0.57 (0.24)*	**1.76 [1.10–2.82]**
Respondent sex (men) × laterality (paternal)	0.37 (0.29)	1.45 [0.83–2.54]
Respondent sex (men) × generation (grandparents)	0.11 (0.22)	1.11 [0.73–1.70]
Respondent sex (men) × sex of relative (men)	−0.01 (0.25)	0.99 [0.60–1.61]
Laterality (paternal) × generation (grandparents)	−0.09 (0.21)	0.91 [0.60–1.38]
Laterality (paternal) × sex of relative (men)	−0.34 (0.22)	0.71 [0.46–1.10]
Generation (grandparents) × sex of relative (men)	0.70 (0.23)**	**2.02 [1.29–3.16]**
*Third-degree relatives and parents’ siblings-in-law*		
Nationality (German/other vs. Austrian)	0.29 (0.18)	1.33 [0.93–1.91]
Respondent sex (men vs. women)	−0.64 (0.27)*	**0.53 [0.31–0.90]**
Laterality (paternal vs. maternal)	−0.07 (0.24)	0.93 [0.58–1.50]
Generation (parents’ siblings-in-law vs. cousin)	−0.86 (0.23)***	**0.42 [0.27–0.67]**
Sex of relative (men vs. women)	−0.77 (0.22)**	**0.46 [0.30–0.72]**
Respondent sex (men) × laterality (paternal)	0.11 (0.30)	1.11 [0.62–2.01]
Respondent sex (men) × generation (parents’ sibling-in-law)	0.38 (0.26)	1.45 [0.87–2.43]
Respondent sex (men) × sex of relative (men)	0.37 (0.24)	1.45 [0.92–2.30]
Laterality (paternal) × generation (parents’ siblings-in-law)	−0.10 (0.25)	0.91 [0.55–1.49]
Laterality (paternal) × sex of relative (men)	0.40 (0.24)	1.50 [0.93–2.40]
Generation (parents’ siblings-in-law) × sex of relative (men)	0.65 (0.26)*	**1.91 [1.15–3.17]**

Only non-redundant model parameters are presented here. With regards to investigated effects, only interactions of substantive interest were included in the models (see main text)

Significant effects (*p* < .05) are printed boldface *p <. 05, **p < .01, ***p < .001

Among medical students (Table 5), second-degree relatives in the medical profession were more likely aunts and uncles than grandparents and, overall, more likely men. This sex-of-relative effect was particularly pronounced among grandparents: Grandfathers were much more likely in the medical profession than grandmothers (generation × sex-of-relative interaction). Among psychology students (Additional file 2: Table S1), similar main effects of generation and sex-of-relative were also present. However, there was no significant (generation) × (sex of relative) interaction. Instead, there was a significant (laterality) × (generation) interaction, such that among psychology students aunts and uncles in the medical profession more likely were from the mother’s than from the father’s side. In addition, psychology students more likely had a second-degree relative in the medical profession when they had other than Austrian nationality.

Among medical students, women reported more third-degree relatives in the medical profession than men (Table 5). Relatives were more likely cousins than parents’ siblings-in-law, and also were more likely women than men. However, this sex effect was confined to cousins; parents’ siblings-in-law in the medical profession did not differ regarding their sex (generation × sex-of-relative interaction). Among psychology students (Additional file 2: Table S1), similar effects, concerning generation and the interaction of (generation) × (sex of relative), were observed (more women among cousins, but not among siblings-in-law). The main effects of respondent sex and generation were not significant.

### Relatives in the psychological and psychotherapeutic professions

Among psychology students, first-degree relatives in the psychological profession and in the psychotherapeutic profession were more likely parents than siblings (Table 6). Additionally, respondents with other than Austrian nationality were more likely to have a first-degree relative in the psychotherapeutic profession, and men were more likely to report a male relative in the psychotherapeutic profession, whereas women were more likely to report a female relative (respondent sex × sex-of-relative interaction). Among medical students, the effect of generation was reversed, i.e., first-degree relatives in the psychological profession more often were siblings than parents (Additional file 2: Table S2). Moreover, there was a sex-of-relative effect, such that first-degree relatives more often were women than men (i.e., sisters and mothers). With regards to first-degree relatives in the psychotherapeutic profession (Additional file 2: Table S3), the effect of generation replicated among medical students. The main effect of nationality and the (respondent sex) × (sex of relative) interaction were not significant.

**Table 6:** Effects of nationality, respondent sex, sex of relative, and generation on the familial aggregation of psychologists and psychotherapists among psychology students for first-degree relatives

Effect	*b* (*SE*)	*OR* [95% confidence interval]
*Psychologists*		
Nationality (German/other vs. Austrian)	0.32 (0.27)	1.37 [0.81–2.33]
Respondent sex (men vs. women)	0.52 (0.58)	1.68 [0.54–5.21]
Generation (parents vs. siblings)	1.03 (0.42)*	**2.81 [1.23–6.41]**
Sex of relative (men vs. women)	−0.70 (0.62)	0.50 [0.15–1.67]
Respondent sex (men) × generation (parents)	−0.55 (0.72)	0.58 [0.14–2.37]
Respondent sex (men) × sex of relative (men)	0.18 (0.97)	1.20 [0.18–7.95]
Generation (parents) × sex of relative (men)	0.08 (0.70)	1.08 [0.27–4.29]
Respondent sex (men) × generation (parents) × sex of relative (men)	−0.27 (1.20)	0.76 [0.07-8.02]
*Psychotherapists*		
Nationality (German/other vs. Austrian)	0.78 (0.32)*	**2.19 [1.17–4.11]**
Respondent sex (men vs. women)	−0.91 (1.15)	0.40 [0.04–3.84]
Generation (parents vs. siblings)	1.96 (0.54)***	**7.09 [2.46–20.45]**
Sex of relative (men vs. women)	−0.81 (0.95)	0.44 [0.07–2.85]
Respondent sex (men) × generation (parents)	0.68 (1.00)	1.97 [0.28–13.99]
Respondent sex (men) × sex of relative (men)	1.81 (0.64)**	**6.08 [1.75–21.15]**
Generation (parents) × sex of relative (men)	−0.94 (1.02)	0.39 [0.05–2.89]

Only non-redundant model parameters are presented here. With regards to investigated effects, only interactions of substantive interest were included in the models (see main text)

Significant effects (*p* < .05) are printed boldface *p <. 05, **p < .01, ***p < .001

### Effects of familial aggregation on respondent age

#### Medical relatives

Among medical students, the main effect of having a first-degree medical relative was significant (Wald-χ^2^(1) = 9.63, *p* = .002), controlling for effects of nationality (Wald-χ^2^(1) = 52.40, *p* < .001) and student sex (Wald-χ^2^(1) = 7.81, *p* = .005; interactions: *p*s ≥ .723; covariate academic age: *p* = .636). Respondents with a first-degree medical relative on average were 0.46 (*SE* = 0.16) years younger than those without. Further, respondents with non-Austrian nationality on average were 1.18 (*SE* = 0.16) years older than Austrians, and men on average were 0.46 (*SE* = 0.16) years older than women. Among psychology students, none of these main effects or interactions reached significance (*p*s ≥ .093), even though the covariate academic age was significant (*p* < .001).

#### Relatives in the psychological or psychotherapeutic professions

In the analyses of relatives in the psychological profession, only the effect of nationality was significant among medical students (Wald-χ^2^(1) = 3.96, *p* = .047; all other tests: *p*s ≥ .071). Respondents with a non-Austrian nationality on average were 1.22 (*SE* = 0.61) years older than Austrians. Among psychology students, only the covariate academic age was significant (*p* < .001; all other tests: *p*s ≥ .054).

In the analyses of relatives in the psychotherapeutic profession, nationality (Wald-χ^2^(1) = 13.59, *p* < .001) and the interaction of (respondent sex) × (first-degree relative) were significant among medical students (Wald-χ^2^(1) = 4.87, *p* = .027; all other tests: *p*s ≥ .496). Again, respondents with a non-Austrian nationality on average were 1.15 (*SE* = 0.31) years older than Austrians. Among men, respondents with a relative in the psychotherapeutic profession were younger than respondents without (mean difference = −0.78 years, *SE* = 0.39, *p* = .046; no significant difference among women, *p* = .219). Among psychology students, nationality (Wald-χ^2^(1) = 6.22, *p* = .013), respondent sex (Wald-χ^2^(1) = 4.38, *p* = .036), first-degree relative (Wald-χ^2^(1) = 12.53, *p* < .001), and the interaction of (nationality) × (first-degree relative) were significant (Wald-χ^2^(1) = 5.10, *p* = .024; covariate academic age: *p* < .001; all other tests: *p*s ≥ .073). Men on average were 0.69 (*SE* = 0.33) years older than women. Overall, non-Austrian students were younger than Austrian students (mean difference = −0.81 years, *SE* = 0.33, *p* = .014), and respondents with a first-degree relative were younger than those without. However, this latter effect was qualified by the nationality factor, such that the effect was only significant for Austrian students (mean difference = −1.90 years, *SE* = 0.60, *p* = .002), whilst not among non-Austrian students (*p* = .114).

## Discussion

This study set out to investigate the familial aggregation of physicians, psychologists, and psychotherapists among first-year students of medicine and psychology in Austria, drawing on nationwide census data of a well-defined cohort. Intriguingly, analogous to Deuteronomy 5:9, these within-family associations of professions indeed can be traced “unto the third generation” (grandparents to grandchildren) and, in the majority, involved male relatives. Familial aggregation effects applied to the whole nation-wide student census in Austria to a similar extent as has been found in previous research (Research Question 1). Differences and temporal trends for Austria are discussed in the two subsequent sections of the Discussion. Familial aggregation of physicians was apparent among both medical students and psychology students and was stronger among the former than the latter group, as was the familial aggregation of psychologists and psychotherapists (see [11]), which was stronger among psychology than among medical students. Thus, familial aggregation effects differed somewhat between medical and psychology students according to the profession of the relatives (Research Question 2), but, overall, generalized to relatives of all three health professions (medicine, psychology, and psychotherapy) (Research Question 3). Implications of the apparent intra-familial ties of these three interrelated disciplines are discussed the fourth section of the Discussion. In contrast to previous results (cf. [5, 6, 9–11]), we found that overall patterns of familial aggregation neither strongly differed between first-, second-, and third-degree relatives, nor between male and female students. Implications of these findings are discussed in the third section of the Discussion. The fifth and sixth section of the Discussion focus on the differences between Austrian and German students and on the age-benefit effects of having a familial background. There, we discuss also implications that may be derived from age-benefit effects on student and career counselling, and on the implementation of study orientation phases and propaedeutics.

### Familial aggregation of physicians

The risk ratios of familial aggregation effects observed in our data were sizable, but among medical students somewhat smaller than previously reported (any medical relative: medical students *RR* = 70 in the present study vs. 109 in [11], psychology students *RR* = 33 vs. 32; any first-degree medical relative: *RR*s = 38 vs. 61 [medical students], 13 vs. 12 [psychology students]). We thus conclude that the familial aggregation of physicians among medical and psychology students is a strong and robust phenomenon in Austria, but may well have diminished somewhat for medical students in recent years, cf. [4, 5]. Whether this decline is a correlate of the ongoing feminization of medicine [20] cannot be fully answered with the present data. Yet, this possibility appears plausible (further evidence for feminization of the medical profession is reviewed below). Alternatively, this difference may have been caused by sample differences between studies, as Voracek et al. [11] investigated a sample of senior medical students. Selective study dropout [2, 4, 5] may have increased the relative number of senior students with a medical background, as compared to the first-year students of the current study. Further data, collected prospectively and longitudinally (e.g., annually), would be necessary to answer this research question more conclusively.

### Familial aggregation of psychologists and psychotherapists

Among psychology students risk ratios increased for relatives in the psychological profession (any relative: *RR* = 49 vs. 38; any first-degree relative: *RR* = 23 vs. 12; see [11]), and remained stable for relatives in the psychotherapeutic profession (any relative: *RR* = 54 vs. 55; any first-degree relative: *RR* = 31 vs. 32). Both the current and previous data [11] stemmed from psychology study beginners. The findings thus suggest an increasing familial aggregation of psychologists among psychology students in Austria in recent years. Psychology and psychotherapy are disciplines that for a long time have been dominated by women (70-80% of psychologists in Austria and Germany are women [35–37]; currently, 72% of all licensed psychotherapists in Austria are women [24]). It appears as though, with familial transmission of medicine losing momentum, familial transmission of psychology gains momentum. Temporal changes of the familial aggregation of psychologists and psychotherapists among medical students could be fruitfully examined in future investigations, as no previous data on these associations are currently available.

### Sex-of-relative and sex-of-student effects

There were notable sex-of-relative effects in our data (see [6, 8, 11]), but only subtle sex-of-student effects. Medical students reported more medical relatives among fathers, grandfathers, and uncles than among mothers, grandmothers or aunts. Within the different degrees of relatedness, it was mostly the elder generation (i.e., parents, grandparents) for which familial aggregation was observed, rather than among the younger generation (i.e., siblings, aunts and uncles; similar results were obtained for psychology and psychotherapy). This accumulation of male and elder-generation medical relatives appears indicative of a cohort effect and the traditional perception of medicine as an allegedly male-typed profession [11, 16, 18, 19]. Yet, our data show that familial aggregation equally applied to male and female students, cf. [6, 8, 11]. A specific male-to-male mode of transmission was observable only for male psychology students who reported more male first-degree relatives (i.e., fathers) in the psychotherapeutic profession than female students. Further, male medical students reported fewer third-degree medical relatives than women, and third-degree relatives in the medical profession were more likely from the same generation as the respondents themselves (i.e., cousins) and more likely female.

The lack of sex-of-student effects, fewer third-degree medical relatives among men, and among third-degree relatives more same-generation and same-sex (i.e., female) relatives jointly point towards temporal changes in the intergenerational transmission of medicine that are consistent with a feminization of the medical profession, as well as with a loosening of intergenerational ties. While traditional preferences motivating sons to pursue the medical profession likely have lessened, given the present data, daughters’ motivation at the same time appears to be influenced by same-sex and same-generation familial peers. Potential causes and consequences of these patterns deserve further scrutiny and need to be followed up in future research. Cross-cultural comparisons would also be informative with regards to these observations.

### Cross-discipline cross-generational effects

The familial aggregation of physicians among psychology students, and of psychologists and psychotherapists among medical students, was substantial, underlining that the professions of medicine, psychology, and psychotherapy are not only interrelated via their various subdisciplines and specialties, but also cross-generationally and within families. Cross-discipline familial associations were mostly similar to same-discipline associations, with some notable exceptions: among psychology students, fathers and mothers were equally likely in the medical profession, and second-degree relatives in the medical profession were specifically more often from the mother’s than from the father’s side. Among medical students, first degree-relatives in the psychological profession were more often siblings than parents, and more often female than male.

Together with the fact that the prevalence of relatives in the medical profession overall was lower among psychology students than among medical students, these patterns suggest that a less male-typed familial perception of medicine may increase the likelihood to pursue an academic study of psychology among individuals with a medical family background. The dominance of women among first-degree relatives in the psychological profession among medical students fits the sex distribution in psychology. Yet, among medical students, ties with psychology appear to be driven more strongly by same-generation relatives in this profession (i.e., sisters), rather than by elder-generation relatives (i.e., mothers). More research is needed here, also concerning possible differences in students’ perception of medicine as a male-typed profession, dependent on the distribution of male and female physicians in their own families.

### Effects of nationality

Familial aggregation appeared stronger among students with a non-Austrian (i.e., mostly German) nationality, and this effect was most clearly visible among psychology students. Inter alia, Germans pursue studies of medicine or psychology in Austria because of strict admission restrictions in Germany [38] (see also [39]) that are based on prior school success (i.e., the numerus clausus system). In Austria, admission to medicine and psychology university studies is solely regulated via admission tests, and neutral with regards to prior school achievement. The observed stronger familial aggregation among non-Austrian students thus underlines the overall importance of a familial background for career and study decisions, which apparently may also raise the likelihood to pursue a study abroad and to pass a required admission test. Of the total German population, only 0.14% are psychologists, 0.06% are psychotherapists (as extrapolated from various personal communications from representatives of the Professional Association of German Psychologists and the German Psychotherapeutic Association), as compared to corresponding figures of 0.29% and 0.16% in Austria. Hence, the risk estimates for the familial aggregation of psychologists and psychotherapists (see Results) likely are downwardly biased for German students.

### Family background and student age

Consistent with previous results on beneficial effects of a family background in medicine [2, 4, 5, 7, 11], the present data are suggestive for age-benefit effects, such that a family background may also lead to earlier career decisions: medical students with a family background were younger than those without. Voracek et al. [11] reported such an age-benefit effect among senior students, particularly for men. In the current study, this effect was observed among both male and female first-year students. Moreover, for the first time we also observed similar effects for relatives in the psychotherapeutic profession: male medical students with a relative in the psychotherapeutic profession were younger than those without, as were Austrian psychology students, regardless of sex. These findings also deserve further investigation, as they indicate that the presence of psychotherapeutic relatives may also bolster study success among medical and psychology students. As for practical implications, this evidence for age-benefit effects could be used for curricular implementation: for example, students with professionally qualified parents (or other relatives) could be specifically recruited as tutors of student-mentoring courses and activities, targetted for those without professional family background.

Apparent age differences between men and women in our data are consistent with the durations of compulsory military or alternative service for men in Austria and Germany up to 2011 (Austria: 6 months military service, 9 months alternative service; Germany: 9 months [6 months in 2011] for both military and alternative service; in 2011, conscription for military service was abolished in Germany). The observed age differences between Austrian and non-Austrian (i.e., mostly German) medical students appear consistent with country differences in the duration of secondary education (9 years in Germany, whereas 8 years in Austria). The 9-year-system was abolished in Germany in the 2000s decade in favor of a 8-year-system. Since the 2010s, some German federal states again have reverted to the 9-year-system. As our data were collected in 2012, the observed age differences were thus likely due to this extra year of secondary education in Germany.

Age differences among psychology students, suggesting a lower age among non-Austrian students, might reflect earlier career decisions among students who pursue this academic study abroad, even in the presence of one extra year of secondary education, as compared with Austrian students. It appears that among Austrians the decision to study psychology on average is made at a later age than the decision to study medicine. In our data, medical relatives were common among both medical and psychology students. This, and the social prestige of medicine [15], could be driving factors for a number of Austrian psychology students to try for admission to medicine in the first place. Having failed admission to medicine may have prompted switching to psychology in this group. This scenario could help explain the age gap between Austrian and non-Austrian psychology students. In addition, some Austrian psychology students may have switched to psychology from related, but non-medical, fields (e.g., education, sociology, or anthropology). If the primary study decision indeed was related to family background, one would expect a stronger familial aggregation of medical than of psychological or psychotherapeutic relatives among students who switched from medicine to psychology, and overall less familial aggregation of medical, psychological, and psychotherapeutic relatives among students who switched from non-medical studies to psychology. Currently, direct evidence supporting these assumptions is lacking. Clearly, more research on these observations is needed.

The current findings do have various implications for student and career counselling, as well as for the implementation of study orientation phases and propaedeutics. Assessing student family background could be of diagnostic value with regards to the motivation and commitment to pursue the study of medicine or psychology, and with regards to prior specialist knowledge of the respective field. Differences in prior specialist knowledge in turn could be beneficially addressed in study orientation phases and propaedeutics, in order to enable students without a professional family background catch-up opportunities. One further point of curricular interest is given through the possibility of family pressure involved in study choice: for instance, high-school graduates conceivably could feel, or actually be, cajoled into medical studies precisely because of an existing family background in medicine. In other words, their study choice would be extrinsically, instead of optimally intrinsically, motivated. Such pathways conceivably could impact on study progress and life satisfaction, as well as on later career decisions and job satisfaction. It would be advantageous to reflect on such family constellations and factors of extrinsic motivation early on, in student counselling and study orientation phases.

### Limitations

Limitations of this study include the absence of direct measures of study success (e.g., exam grades). Hence, effects of familial aggregation on study success could not be investigated. Likewise, respondents’ prior academic studies were not queried. Therefore, switching from medicine to psychology or vice versa, or from any other academic study to the studies of medicine and psychology, could not be examined.

We analyzed the effects of familial aggregation with regards to type of relative, not accounting for the total numbers of relatives (within or across the types of relatives) in the profession of interest. This may have impacted on our results.

Admission regulations, rates, and tests, and fees for the academic studies of medicine and psychology vary widely between, but also within, countries. Also, the relatively high numbers of foreign (predominantly German) students in the academic studies of medicine and psychology in Austria is only comparable to the situation in some, by no means all, European or non-European countries. Finally, only students of public universities were in the focus of the current study. Hence, the present results may not readily generalize to other countries or to the sector of private universities.

## Conclusions

This study provided a representative and detailed look into the familial aggregation of physicians, psychologists, and psychotherapist among an entire cohort of first-year students of medicine and psychology in Austria. We found that the familial aggregation of all three professions overall is high in these two disciplines. Previous findings regarding the familial aggregation of physicians among medical students therefore apparently generalize to relatives from the three related health professions for both medical and psychology students. Compared to prior related evidence from Austria, obtained trends are consistent with notions of a feminization of medicine and a small decline of the familial aggregation of physicians among medical students in recent years. At the same time, the familial aggregation of psychologists increased among psychology students. Both the medical and the psychology curricula have been entirely restructured and coupled with entrance examinations and limited numbers of study places at all public universities in Austria during the elapsed years. Still, familial aggregation remained quite stable and sizeable, or even increased, as in the case of psychology. Further, a family background in medicine exerted age-benefit effects for medical students, as did a family background in psychotherapy for psychology students. These constellations apparently brought about the decision to study medicine or psychology at an earlier age. Our results thus indicate that professional family backgrounds need to be considered in investigations on study and career decisions among medical and psychology undergraduates. Assessing the family background may also be of diagnostic value for student and career counselling. Benefits of familial aggregation may further need to be considered, or could be fruitfully utilized, for curricular implementation. For example, study orientation phases and propaedeutics could be tailored to specifically support students without a professional family background to catch up and to fill potential gaps in specialist knowledge of the field. Students with a professional family background could provide support in the context of student-mentoring offerings.

Proximate and distal causes and consequences of the temporal changes of familial aggregation observed in our data, compared to previous results, need to be followed up in the future. Cross-cultural comparisons, but more specifically also comparisons with regards to different academic admission systems, school systems, and legal regulations of the medical, psychological, and psychotherapeutic professions in different countries, are needed.

## Additional files

Additional file 1: Survey form. (PDF 372 kb)

Additional file 2: Tables S1 to S3. (DOCX 23 kb)
