# Supplementary material for: Unto the third generation: evidence for strong familial aggregation of physicians, psychologists, and psychotherapists among first-year medical and psychology students in a nationwide Austrian cohort census
Source: BMC Med Educ. 2017 May 3;17:81. doi: 10.1186/s12909-017-0921-4 (PMC5415715; doi:10.1186/s12909-017-0921-4)
Supplement: Supplementary file 1 — Survey form. (PDF 372 kb) [file 12909_2017_921_MOESM1_ESM.pdf]

Studie: Psychosoziale und Gesundheitsberufe in Familien

Sehr geehrte Studierende—Liebe KollegInnen: Wir ersuchen Sie um Ihre Teilnahme an der vorliegenden Erhebung (DA Nina Berger, Fak. f. Psychologie, Univ. Wien). Dieser Fragebogen (1 Seite) ist selbstleitend, **anonym** und dauert etwa **3-5 Minuten**.

Herzlichen Dank im Voraus für Ihre Teilnahme!

Geschlecht: ◻ weiblich ◻ männlich Alter: ________ Jahre

Staatsangehörigkeit: ___________________ Ich bin derzeit im ___. **Studiensemester**

Bitte geben Sie in der Tabelle durch entsprechendes Ankreuzen an (bei Mehrfachfällen: bitte jeweils Anzahl angeben), wenn Familienmitglieder von Ihnen

- entweder das Studium der Psychologie oder der Humanmedizin oder eine Psychotherapie-Ausbildung abgeschlossen haben (bei Humanmedizin bitte zusätzlich um Angabe des ärztlichen Faches)
- oder Psychologie oder Humanmedizin studieren oder in Psychotherapie-Ausbildung sind.

Verwandte(r)	Psychologie	Psychotherapie	Humanmedizin	Medizinisches Fach
Schwester (auch: Halbschwester)	◻	◻	◻	
Bruder (auch: Halbbruder)	◻	◻	◻	
meine Mutter	◻	◻	◻	
mein Vater	◻	◻	◻	
Mütterlicherseits				
meine Großmutter (Mutterseite)	◻	◻	◻	
mein Großvater (Mutterseite)	◻	◻	◻	
Tante (Schwester meiner Mutter)	◻	◻	◻	
Onkel (Bruder meiner Mutter)	◻	◻	◻	
angeheiratete Tante (Mutterseite)	◻	◻	◻	
angeheirateter Onkel (Mutterseite)	◻	◻	◻	
Cousine (Nichte meiner Mutter)	◻	◻	◻	
Cousin (Neffe meiner Mutter)	◻	◻	◻	
Väterlicherseits				
meine Großmutter (Vaterseite)	◻	◻	◻	
mein Großvater (Vaterseite)	◻	◻	◻	
Tante (Schwester meines Vaters)	◻	◻	◻	
Onkel (Bruder meines Vaters)	◻	◻	◻	
angeheiratete Tante (Vaterseite)	◻	◻	◻	
angeheirateter Onkel (Vaterseite)	◻	◻	◻	
Cousine (Nichte meines Vaters)	◻	◻	◻	
Cousin (Neffe meines Vaters)	◻	◻	◻	

Bei Interesse an den Ergebnissen dieser Studie bitte Mail an **Familienstudie-Uni-Wien@gmx.at**
