# Supplementary material for: Unto the third generation: evidence for strong familial aggregation of physicians, psychologists, and psychotherapists among first-year medical and psychology students in a nationwide Austrian cohort census
Source: BMC Med Educ. 2017 May 3;17:81. doi: 10.1186/s12909-017-0921-4 (PMC5415715; doi:10.1186/s12909-017-0921-4)
Supplement: Supplementary file 2 — Tables S1 to S3. (DOCX 23 kb) [file 12909_2017_921_MOESM2_ESM.docx]

**Table S1.** Effects of nationality, respondent sex, sex of relative, generation, and laterality (among second-degree and third-degree relatives) on the familial aggregation of physicians among psychology students, separately for first-degree, second-degree, and third-degree relatives (includes parents’ siblings-in-law)

| Effect | *b* (*SE*) | *OR* [95% confidence interval] |
| --- | --- | --- |
| *First-degree relatives* | | |
| Nationality (German/other vs. Austrian) | 0.25 (0.24) | 1.28 [0.80–2.07] |
| Respondent sex (men vs. women) | 0.14 (0.69) | 1.15 [0.30–4.46] |
| Generation (parents vs. siblings) | **0.90 (0.46)*** | **2.47 [1.01–6.03]** |
| Sex of relative (men vs. women) | 0.55 (0.48) | 1.73 [0.67–4.45] |
| Respondent sex (men) × generation (parents) | -0.61 (0.68) | 0.54 [0.14–2.07] |
| Respondent sex (men) × sex of relative (men) | 0.46 (0.84) | 1.58 [0.30–8.21] |
| Generation (parents) × sex of relative (men) | 0.20 (0.57) | 1.23 [0.41–3.72] |
| Respondent sex (men) × generation (parents) × sex of relative (men) | 0.01 (0.85) | 1.01 [0.19-5.33] |
| *Second-degree relatives* | | |
| Nationality (German/other vs. Austrian) | **0.51 (0.23)** | **1.66 [1.06–2.59]** |
| Respondent sex (men vs. women) | -0.02 (0.48) | 0.99 [0.38–2.53] |
| Laterality (paternal vs. maternal) | -0.56 (0.38) | 0.60 [0.27–1.20] |
| Generation (grandparents vs. aunts/uncles) | **-1.15 (0.39)**** | **0.32 [0.15–0.68]** |
| Sex of relative (men vs. women) | **0.56 (0.25)*** | **1.75 [1.06–2.88]** |
| Respondent sex (men) × laterality (paternal) | -0.52 (0.57) | 0.60 [0.20–1.81] |
| Respondent sex (men) × generation (grandparents) | -0.06 (0.45) | 0.94 [0.39–2.27] |
| Respondent sex (men) × sex of relative (men) | -0.08 (0.38) | 0.92 [0.44–1.94] |
| Laterality (paternal) × generation (grandparents) | **0.80 (0.40)*** | **2.23 [1.02–4.89]** |
| Laterality (paternal) × sex of relative (men) | -0.22 (0.38) | 0.80 [0.38–1.70] |
| Generation (grandparents) × sex of relative (men) | 0.03 (0.40) | 1.03 [0.47–2.27] |
| *Third-degree relatives and parents’ siblings-in-law* | | |
| Nationality (German/other vs. Austrian) | 0.21 (0.22) | 1.24 [0.80–1.91] |
| Respondent sex (men vs. women) | -0.21 (0.40) | 0.81 [0.37–1.79] |
| Laterality (paternal vs. maternal) | -0.44 (0.32) | 0.64 [0.35–1.20] |
| Generation (parents’ siblings-in-law vs. cousin) | **-1.03 (0.32)***** | **0.36 [0.19–0.67]** |
| Sex of relative (men vs. women) | -0.26 (0.27) | 0.77 [0.46–1.30] |
| Respondent sex (men) × laterality (paternal) | -0.11 (0.50) | 0.90 [0.34–2.38] |
| Respondent sex (men) × generation (parents’ sibling-in-law) | 0.11 (0.46) | 1.12 [0.45–2.78] |
| Respondent sex (men) × sex of relative (men) | -0.47 (0.48) | 0.63 [0.25–1.60] |
| Laterality (paternal) × generation (parents’ siblings-in-law) | 0.55 (0.42) | 1.73 [0.77–3.89] |
| Laterality (paternal) × sex of relative (men) | -0.57 (0.38) | 0.56 [0.27–1.19] |
| Generation (parents’ siblings-in-law) × sex of relative (men) | **0.94 (0.33)**** | **2.57 [1.33–4.95]** |

*Note*. Only non-redundant model parameters are presented here. With regards to investigated effects, only interactions of substantive interest were included in the models (see main text).

**Table S2.** Effects of nationality, respondent sex, sex of relative, and generation on the familial aggregation of psychologists among medical students for first-degree relatives

| Effect | *b* (*SE*) | *OR* [95% confidence interval] |
| --- | --- | --- |
| Nationality (German/other vs. Austrian) | -0.17 (0.41) | 0.84 [0.38–1.89] |
| Respondent sex (men vs. women) | -0.003 (0.44) | 1.00 [0.42–2.36] |
| Generation (parents vs. siblings) | **-1.88 (0.82)*** | **0.15 [0.03–0.76]** |
| Sex of relative (men vs. women) | **-2.92 (1.20)*** | **0.05 [0.01–0.57]** |
| Respondent sex (men) × generation (parents) | 1.54 (0.92) | 4.67 [0.78–28.11] |
| Respondent sex (men) × sex of relative (men) | 1.33 (1.13) | 3.79 [0.41–35.20] |
| Generation (parents) × sex of relative (men) | 0.79 (0.77) | 2.20 [0.49–9.96] |

*Note*. Only non-redundant model parameters are presented here. With regards to investigated effects, only interactions of substantive interest were included in the models (see main text).

**Table S3.** Effects of nationality, respondent sex, sex of relative, and generation on the familial aggregation of psychotherapists among medical students for first-degree relatives

| Effect | *b* (*SE*) | *OR* [95% confidence interval] |
| --- | --- | --- |
| Nationality (German/other vs. Austrian) | 0.11 (0.37) | 1.12 [0.54–2.32] |
| Respondent sex (men vs. women) | -0.59 (0.53) | 0.56 [0.20–1.56] |
| Generation (parents vs. siblings) | **2.71 (0.73)***** | **15.07 [3.57–63.60]** |
| Sex of relative (men vs. women) | -0.34 (0.49) | 0.72 [0.28–1.86] |
| Respondent sex (men) × sex of relative (men) | 1.14 (0.67) | 3.11 [0.84–11.59] |

*Note*. Only non-redundant model parameters are presented here. With regards to investigated effects, only interactions of substantive interest were included in the models (see main text).
